# Partial Priapism Treated with Pentoxifylline

**DOI:** 10.1590/S1677-5538.IBJU.2014.0363

**Published:** 2015

**Authors:** Meghan A. Cooper, Rafael E. Carrion, Christopher Yang

**Affiliations:** 1 Lake Erie College of Osteopathic Medicine, Bradenton, Florida, USA; 2 Department of Urology, University of South Florida Morsani College of Medicine, Tampa, Florida, USA

**Keywords:** Priapism, Penis, Penile Induration, Erectile Dysfunction

## Abstract

**Main findings:**

A 26-year-old man suffering from partial priapism was successfully treated with a regimen including pentoxifylline, a nonspecific phosphodiesterase inhibitor that is often used to conservatively treat Peyronie's disease.

**Case hypothesis:**

Partial priapism is an extremely rare urological condition that is characterized by thrombosis within the proximal segment of a single corpus cavernosum. There have only been 36 reported cases to date. Although several factors have been associated with this unusual disorder, such as trauma or bicycle riding, the etiology is still not completely understood. Treatment is usually conservative and consists of a non-steroidal anti-inflammatory and anti-thrombotic.

**Promising future implications:**

This case report supports the utilization of pentoxifylline in patients with partial priapism due to its anti-fibrogenic and anti-thrombotic properties.

## SCENARIO

A 26-year-old man presented to the emergency department with severe, right-sided perineal pain of 24 hours duration. He described the pain as non-radiating and sharp, denied any trauma, but did have mild dysuria. The patient had chlamydial urethritis one year ago that was successfully treated with oral antibiotics. His last reported sexual encounter was three months ago, and he was an avid bicycle motocross rider. He denied tobacco or marijuana use, but did use alcohol several hours prior to initial presentation of pain. Physical examination was remarkable only for a 2cm tender area of firm induration on the right aspect of the perineum.

**Figure 1:**
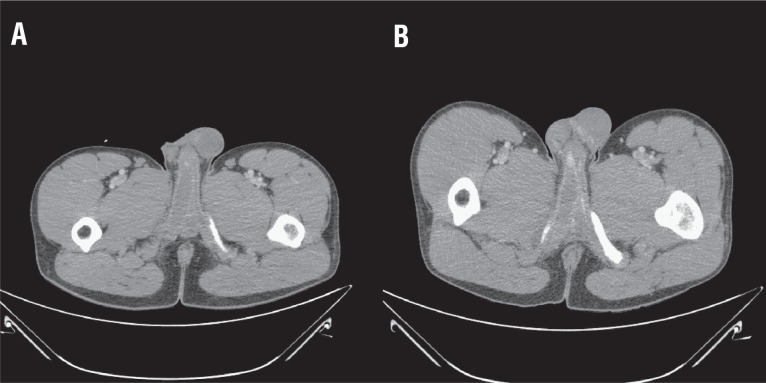
Computed tomography image of patient with partial priapism of the proximal right corpus cavernosum. A) Initial imaging at first presentation to the emergency room; B) Imaging three days later showing well-defined transverse membrane separating the proximal thrombosed and distal non-thrombosed sections of right corpus cavernosum.

Complete blood count, comprehensive metabolic panel, and urinalysis were unremarkable, with the exception of urinary nitrites and urine urobilinogen of 2.0mg/dL. Computed tomography (CT) of the pelvis showed asymmetric enlargement of the proximal right corpus cavernosum with an area of thin hyperdense tissue distal to the enlarged region (Figure-1A). The patient was evaluated by a urology resident, discussed with an attending urologist, and discharged with ciprofloxacin for presumed urinary tract infection.

Three days later he returned to the hospital with worsening right-sided perineal pain. In the interim he had erections that exacerbated the pain, and detumescence did not result in resolution of the pain or perineal swelling. Physical examination was unchanged. Repeat laboratory tests showed normalization of all parameters. Repeat CT showed the same picture, with a hyperdense membrane distal to the right proximal corporal thrombosis (Figure-1B). He was evaluated by a different urology resident and attending physician, and diagnosed with partial priapism. He was discharged with conservative treatment of ibuprofen 800mg three times daily, acetylsalicylic acid 325mg daily, and pentoxifylline 400mg twice daily.

At follow-up two days later he had significantly decreased pain, though still had right proximal cavernosal swelling and tenderness to palpation. The pain and perineal swelling completely resolved over the course of one month. He stopped taking the prescribed medications six weeks after initial presentation. At last follow-up three months after initial presentation, the patient reported continued resolution of perineal swelling, no impairment of erectile function and no further episodes of partial priapism.

### Case hypothesis and rationale

Partial priapism is a rare non-emergent condition first reported in 1976 (1, 2). The typical presentation is a young man with a firm, painful perineal mass sometimes accompanied by erectile dysfunction, penile pain, dysuria, or decreased urinary flow (3, 4). Several factors have been associated with partial priapism, including bicycle riding, trauma, prolonged or vigorous intercourse, hematologic disorders, history of prostatitis or hepatitis A, marijuana and cocaine abuse, use of tamsulosin or sildenafil, recent airplane flight, prior priapism, and fever of unknown origin (3, 4). Initial treatment for partial priapism is conservative, consisting of non-steroidal anti-inflammatory drugs (NSAIDs), acetylsalicylic acid, heparin, or low molecular weight heparin. In cases not responding to conservative therapy, surgical intervention is required (3–5).

This report describes the first known usage of pentoxifylline (PTX) in the treatment of partial priapism. This also highlights the need for greater awareness of partial priapism as a formal pathologic entity in the medical community.

### Discussion and future perspectives

Partial priapism is an uncommon condition characterized by thrombosis within the proximal segment of the corpus cavernosum. There have been only 36 cases reported previously in the world literature (4, 6). The initial cases of partial priapism were treated surgically with corporotomy and clot evacuation that led to a high rate of recurrence and erectile dysfunction (7). Recently most cases are treated conservatively with NSAIDs, acetylsalicylic acid, and/or anticoagulants (3, 4, 8).

The etiology of this condition remains elusive. Ilicki and colleagues proposed a two-hit model to explain the mechanism of partial segmental thrombosis in which a permeable transverse membrane divides the corpus cavernosum into a proximal and distal portion. This membrane can be either congenital or posttraumatic. Secondly, there must be a triggering event, often trauma to the penis, which blocks the permeable parts of the membrane. With tumescence the intracorporal blood can form clot, which impedes the membrane even more. This results in thrombosis of the proximal corpus cavernosum, thereby causing partial priapism (3).

This model suggests a role for PTX in the treatment of partial priapism. PTX is a nonspecific phosphodiesterase inhibitor that has been used in a variety of fibrotic conditions, including Peyronie's disease (PD), radiation proctitis, radiation-induced fibrosis, alcoholic hepatitis, steatohepatitis, cystic fibrosis, radiation pneumonitis, epidural fibrosis, osteo-radionecrosis, and to prevent atherosclerosis in hypertensive patients with type 2 diabetes mellitus (9, 10). The role of PTX as an anti-fibrotic is familiar to urologists in its use in the treatment of PD, where it has been shown to potentially decrease penile curvature and plaque volume through its inhibitory effects on fibrogenesis (9–11). As a nonselective phosphodiesterase inhibitor, PTX decreases tumor necrosis factor-alpha release and nuclear factor-kappa-B transcription, thereby decreasing collagen production (11). PTX also attenuates transforming growth factor-beta, which has been implicated in the pathogenesis of numerous human fibrotic disorders, through its role in stimulating collagen deposition and mediating plaque formation (11).

PTX has also been investigated in the treatment of erectile dysfunction (12). Pathologically, the loss of corporal smooth muscle cells and excess collagen deposition can lead to erectile dysfunction (13). PTX up-regulates nitric oxide, which leads to the destruction of reactive oxygen species, thereby mitigating tissue inflammation and fibrosis. In addition, PTX can improve blood flow by decreasing viscosity, increasing red blood cell flexibility and inhibiting thrombocyte aggregation (12, 13).

For patients with partial priapism, PTX may be a treatment option due to its effect on both components of Ilicki's two-hit theory. The anti-fibrotic properties of PTX may improve the permeability of the intracorporal membrane, and the improvement in blood flow and inhibited thrombocyte aggregation may improve flow through the membrane.

The present patient had a transverse membrane separating the thrombosed and non-thrombosed sections of the proximal right corpus cavernosum (Figure-1). He was treated with the most commonly-described conservative management (NSAIDs and acetylsalicylic acid), as well as PTX. It is unknown whether the addition of PTX to the previously-described regimen hastened or hindered his return to normal function. However, as he returned to full function within one month of initiation of treatment, it is unlikely that PTX significantly worsened his recovery. Further investigation would be beneficial for verification of the efficacy of PTX in partial priapism, but the extreme scarcity of patients presenting with this condition precludes randomized controlled trials.

Finally, the management of our patient was less than ideal due to lack of recognition of the disease. He presented to the emergency room in two separate times within a three day span and was evaluated by our urology service each time. As he had some symptoms and laboratory abnormalities similar to urinary tract infection, he was discharged after the first evaluation with antibiotics despite CT showing the pathognomonic picture of partial priapism. Prior to this case, none of the urologists at our institution had ever treated a case of partial priapism. With this patient being only the 37th reported case of the disease, it is likely that the vast majority of urologists have never treated or even have knowledge of this disease. With this and additional reports, it is our hope that physicians will recognize partial priapism with higher diagnostic acumen.

In conclusion, partial priapism is a rare, non-emergent urological condition that is typically treated conservatively with NSAIDs and acetylsalicylic acid. This is the first case where PTX was added to the conventional treatment regimen, with excellent clinical response. Though further investigation is required to verify efficacy, in the rare case of partial priapism the use of PTX may be considered.

## Abbreviations

CT =: computed tomography
NSAIDs =: non-steroidal anti-inflammatory drugs
PD =: Peyronie's disease
PTX =: pentoxifylline
